# Microglial displacement of inhibitory synapses provides neuroprotection in the adult brain

**DOI:** 10.1038/ncomms5486

**Published:** 2014-07-22

**Authors:** Zhihong Chen, Walid Jalabi, Weiwei Hu, Hyun-Joo Park, John T. Gale, Grahame J. Kidd, Rodica Bernatowicz, Zachary C. Gossman, Jacqueline T. Chen, Ranjan Dutta, Bruce D. Trapp

**Affiliations:** 1 Department of Neurosciences, Lerner Research Institute, Cleveland Clinic, Cleveland, Ohio 44195, USA; 2 Department of Pharmacology, School of Basic Medical Sciences, Zhejiang University, Hangzhou, Zhejiang 310058, China; 3 Center for Neurological Restoration, Neurological Institute, Cleveland Clinic, Cleveland, Ohio 44195, USA; 4 These authors contributed equally to this work

## Abstract

Microglia actively survey the brain microenvironment and play essential roles in sculpting synaptic connections during brain development. While microglial functions in the adult brain are less clear, activated microglia can closely appose neuronal cell bodies and displace axosomatic presynaptic terminals. Microglia-mediated stripping of presynaptic terminals is considered neuroprotective, but the cellular and molecular mechanisms are poorly defined. Using 3D electron microscopy, we demonstrate that activated microglia displace inhibitory presynaptic terminals from cortical neurons in adult mice. Electrophysiological recordings further establish that the reduction in inhibitory GABAergic synapses increased synchronized firing of cortical neurons in γ-frequency band. Increased neuronal activity results in the calcium-mediated activation of CaM kinase IV, phosphorylation of CREB, increased expression of antiapoptotic and neurotrophic molecules and reduced apoptosis of cortical neurons following injury. These results indicate that activated microglia can protect the adult brain by migrating to inhibitory synapses and displacing them from cortical neurons.

## Abstract (web summary)

Microglia play essential roles in sculpting synaptic connections during brain development but their role in the adult brain is less clear. Here the authors show that activated microglia can prophylactically protect the adult rodent brain from injury by migrating to and displacing inhibitory synapses from cortical neurons.

## Graphical abstract

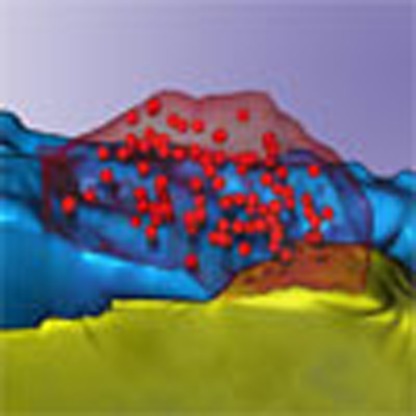

Microglia are the resident immune cells of the central nervous system (CNS) and the primary responders in a defence network that covers the entire brain parenchyma. Microglia have at least two functions that are common to monocytic innate immune cells in all organs: they kill and phagocytize viruses, bacteria and other foreign invaders and they scavenge cellular debris as part of wound healing and tissue repair processes^1^. In addition to these classical innate immune responses, recent studies have identified an essential role for microglia in shaping synaptic fields during postnatal brain development^2^,^3^. The roles of microglial synaptic modulation in the adult brain are less clear. Microglia are activated by acute insults and chronic disease states. This results in enlargement of their cell bodies, asymmetrical distribution of their processes and increased expression of activation molecules^1^. Whether microglial activation is protective or destructive to the CNS remains controversial^4^. Historically, most studies have focused on a destructive role for microglia in the adult brain. Since it is difficult to distinguish destructive and phagocytic microglial phenotypes, there is little direct evidence that microglia are primary mediators of brain damage *in vivo*^5^,^6^. While recent studies support the concept that microglial activation can protect the adult brain^7^,^8^,^9^, the precise cellular and molecular mechanisms of this microglial neuroprotection remain to be established.

Intravital imaging has shown that microglial processes are highly motile even under ‘resting’ physiological conditions^10^. As microglial processes probe their microenvironment, they associate with and sense the activity of individual synapses^11^,^12^. The superfluous excitatory synapses overproduced during brain development are eliminated by the microglia^2^. This microglia-mediated phagocytosis of excitatory synapses depends on the reduced electrical activity of the synapses, the expression of the complement cascade component C1q by neurons and the expression of the complement receptor 3 (CD11b/CD18) by microglia^13^,^14^. In addition to synaptic elimination during development, microglia-mediated synaptic stripping has been reported in the adult CNS^15^. Following transection of the mouse facial nerve, microglia in the facial nucleus become activated, physically ensheath injured motor neurons and displace axosomatic synapses^15^. This ‘synaptic stripping’ was associated with motor neuron regeneration and considered to be neuroprotective^16^. Microglial displacement of inhibitory synapses from cortical neurons has also been associated with a transient preconditioning paradigm that protects the brain from subsequent injury^8^.

A candidate mechanism by which microglia-mediated synaptic modulation imparts neuroprotection in the adult brain is the reduction of inhibitory synaptic input on neuronal cell bodies. Among the best documented neuroprotective mechanisms is the activation of synaptic *N*-methyl *D*-aspartate (NMDA) glutamate receptors^17^. During brain development, neurons are overproduced and only those that make and receive appropriate synaptic connections survive. Increased activation of neuronal synaptic NMDA receptors (NMDARs) can also be induced by the GABA_A_ receptor antagonist bicuculline^18^. Inhibition of GABAergic activity increases the firing of synaptic NMDARs, which induces phosphorylation of the transcription factor CREB (cAMP responses element binding protein) and *de novo* transcription of genes that promote neuronal survival^19^,^20^. Therefore, microglia-mediated removal of inhibitory synapses may also induce neuroprotection by increasing synchronic neuronal firing and associated calcium transient-mediated prosurvival gene transcription.

We have previously shown that systemic administration of low-dose lipopolysaccharide (LPS), a TLR4 agonist, globally activates microglia and protects the brain from subsequent injury^8^. These activated microglia physically surround the cell bodies of cortical projection neurons. Following challenge with a traumatic brain injury (TBI), this LPS preconditioning paradigm reduced neuronal apoptosis and lesion volume. In the present study, we further investigated (1) whether activated microglia remove inhibitory synapses from neuronal perikarya, and if so, whether this loss of inhibition results in synchronized neuronal activity and (2) the molecular mechanisms by which LPS activation of microglia induces neuroprotection in the adult brain. Using three-dimensional electron microscopy (3D-EM), we provide evidence that activated microglia displace axosomatic inhibitory synapses from cortical neurons. Electrophysiological recordings demonstrate that the reduction in inhibitory GABAergic synapses increases synchronized neuronal activity, which underlies the calcium-mediated activation of CaM kinase IV, phosphorylation of CREB, upregulated expression of antiapoptotic and neurotrophic molecules and reduced apoptosis of cortical neurons following injury. Collectively, these observations support a neuroprotective role for activated microglia in the adult brain and begin to unravel the cellular and molecular mechanisms by which microglia-mediated synaptic displacement induces neuroprotection.

## Results

### Activated microglia displace inhibitory GABAergic synapses

**Figure 1:**
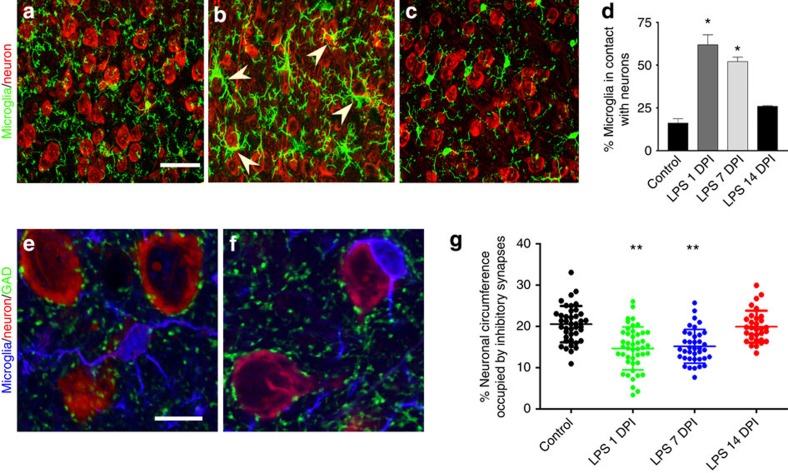
Activated microglia target cortical neurons and displace inhibitory synapses from neuronal soma. (**a**–**c**) Confocal micrographs show morphological changes of microglia (green) following multiple LPS injections. Arrowheads indicate microglia enwrapping neuronal cell bodies (red). Scale bar, 40 μm. (**d**) Quantification of the percentage of microglia in contact with neurons. *N*=3 per group. (**e**,**f**) Triple labelling of microglia (blue), neurons (red) and GAD^+^ inhibitory synapses (green). Scale bar, 10 μm. (**g**) Quantification of the percentage of neuronal circumference occupied by inhibitory synapses over the indicated time course. *N*=31 to 55 neurons sampled from six individual mice per group. Error bars represent s.e.m. *P<0.05; **P<0.01 compared with control group using a one-way ANOVA test.

Intraperitoneal administration of a low dose of LPS (1.0 mg kg^−1^) for four consecutive days results in global microglial activation in the brain parenchyma and these activated microglia closely appose neuronal perikarya^8^. Microglial activation is defined by hallmark morphological changes and the upregulation of signature activation markers such as F4/80. Here, we further characterized this activation by quantifying the percentage of total microglia in close apposition with neuronal soma in laminar layers III to V in motor cortices of mice. The temporal changes of this association were followed for 2 weeks after the final LPS or saline injection (Fig. 1; Supplementary Fig. 1). Resting microglia in saline-injected mice had a stellate appearance and no obvious association with neuronal somata (Fig. 1a). Sixteen per cent of these cortical microglia extended processes that closely apposed but never ensheathed neuronal somata or apical dendrites (Fig. 1d). In contrast, 1 day after the final LPS injection (1 day post injection; 1 DPI), 61% of microglia ensheathed neuronal perikarya or extended asymmetrically oriented processes that ensheathed neurons (Fig. 1b,d, arrowheads). These LPS-induced microglial changes were transient, as microglial activation (Supplementary Fig. 1) and their neuronal associations were reduced to 50% at 7 DPI (Fig. 1d), and microglia displayed a resting state morphology at 14 DPI (Fig. 1c,d).

The majority of synapses terminating on projection neuronal soma in the cerebral cortex are GABAergic inhibitory synapses^21^. To investigate whether the close apposition of neurons by activated microglia displaces inhibitory synaptic terminals, we quantified the neuronal cell body circumference occupied by inhibitory presynaptic terminals in cortical layers III and V of PBS- (Fig. 1e) and LPS-injected mice (Fig. 1f). Sections were triple labelled with anti-GAD 65/67 (glutamic acid decarboxylase 65/67) to identify GABAergic presynaptic terminals, anti-Iba-1 for microglia and Nissl for neurons. In control mice, 20.5% of the neuronal circumference was occupied by GAD^+^ terminals, consistent with previously published results^21^. One day after the final LPS injection, the coverage of neuronal surface by GAD^+^ punctae was reduced to 14.7%, representing a 27% decrease compared with PBS-injected mice (Fig. 1g). In LPS-treated mice, the neuronal circumference occupied by GAD-positive presynaptic terminals was partially recovered at 7 DPI (15.2%) and was similar to control levels at 14 DPI (19.9%), indicating that neuronal cell bodies re-establish inhibitory synaptic contacts after microglia have returned to their resting state. These data establish that activated microglia transiently displace inhibitory synaptic input to cortical projection neurons following four daily LPS injections.

### Activated microglia displace axosomatic synapses

While confocal micrographs indicate that microglial cells displace inhibitory presynaptic terminals from neuronal perikarya (Fig. 1f), it is not known whether these activated microglia actively ‘strip’ presynaptic structures from the neuronal surface or if they play a secondary role of ensheathing neurons in response to presynaptic terminal withdrawal. We investigated these possibilities using 3D-EM^22^ to trace cellular structures in three dimensions at nanometre resolution.

**Figure 2:**
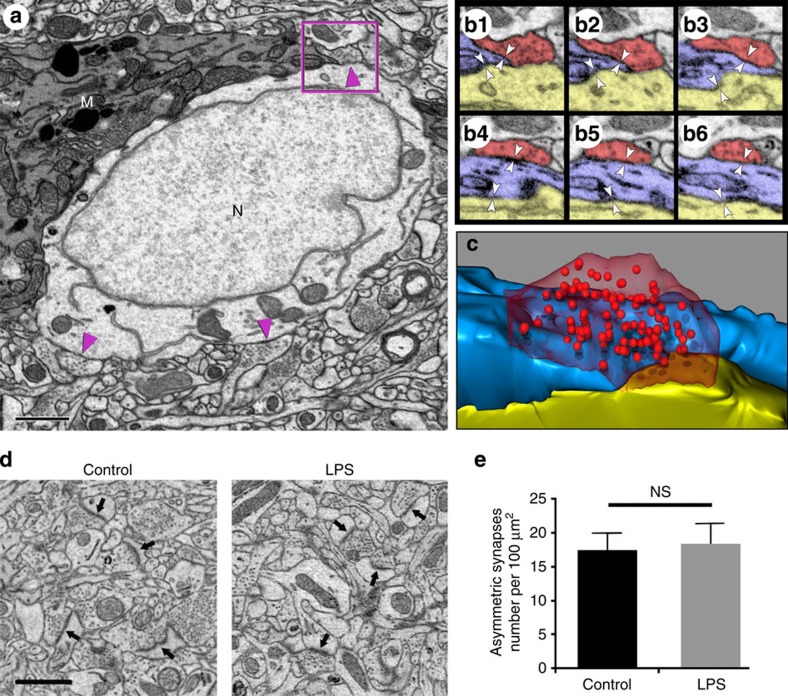
3D-EM reveals axosomatic synaptic displacement by activated microglia. (**a**) Electron micrograph of a microglia (‘M’) closely apposing part of a neuronal soma (‘N’) 24 h after the final LPS injection. Arrowheads indicate axosomatic inhibitory synapses. A region of interest is outlined in the box in **a** and serial sections of this region are shown in **b**. Scale bar, 1 μm. (**b**) In these serial EM sections, the leading edge of the microglial process (blue) partially displaces the presynaptic terminal (red), while the remainder of this terminal retains a normal synaptic cleft with the neuronal perikarya (yellow). Pairs of arrowheads indicate close contact between the microglia, the neuron and the synaptic terminal. (**c**) 3D reconstruction of the serial images shown in **b**. (**d**) Representative electron micrographs of motor cortex neuropil from PBS- and LPS-injected mice. Arrows point to asymmetric excitatory synapses. Scale bar, 1 μm. (**e**) The densities of asymmetrical excitatory synapses were similar in the motor cortices from PBS- and LPS-injected mice. NS, not significant; *t*-test.

Laminar layers III to V of frontal lobe motor cortices of PBS- or LPS-treated mice were processed and stacks of consecutive electron micrographs were obtained using a Zeiss 3D-scanning electron microscope equipped with a Gatan in-chamber ultramicrotome^23^. Microglia were identified by their characteristic electron-dense cytoplasm containing conspicuous inclusion bodies, elongated mitochondria and late lysosomes^24^. A representative image (Fig. 2a) shows an activated microglia (‘M’) closely apposing part of a neuronal soma 24 h after the final LPS injection. Where the microglia is absent, presynaptic boutons with abundant synaptic vesicles are distributed around the neuronal surface membrane containing symmetrical post-synaptic densities, indicative of inhibitory synapses^25^ (Fig. 2a, arrowheads). Conversely, where the microglia tightly apposes the neuronal perikarya (‘N’), presynaptic structures are not present.

One of the possible outcomes of 3D-EM analysis is the ability to detect microglial cells caught in the act of physically displacing a synapse from the neuronal surface. Such a region of interest is outlined by the box in Fig. 2a and a series of images of this region are enlarged (Fig. 2b), showing the neuron (yellow), microglia (blue) and the presynaptic terminal (red). The leading edge of this microglial process partially displaced the presynaptic terminal, while the remainder of this terminal retains a normal synaptic cleft with the neuronal perikarya (Fig. 2b1). The microglia closely apposes both the neuronal plasma membrane and the displaced presynaptic terminal (pairs of arrowheads in Fig. 2b). The relationship between the microglial process, the presynaptic terminal and the neuronal surface is schematically reconstructed in three dimensions in Fig. 2c. Approximately, 90% of the presynaptic terminal is detached from the neuronal surface, while 10% maintains a normal synaptic cleft with the neuron.

The 3D-EM data described thus far establish that neuronal surfaces with microglial attachment have lost inhibitory synaptic input. However, is it also possible that inhibitory synaptic terminals withdraw from the soma without microglial insertion? To address this question, we randomly selected micrographs of 15 cortical motor neurons from either PBS- (*n*=3) or LPS-injected mice (*n*=3) that had no contact with activated microglia. If synaptic terminals withdraw from the soma without microglial insertion, we would expect to observe presynaptic terminals detached from the neuronal surface or ‘orphaned’ inhibitory synapses (that is, presynaptic terminals without a receptive post-synaptic terminal) around these neurons despite their lack of association with activated microglia. We found no differences in neuronal diameter, number of synapses per neuron or the length of active zones (where synaptic vesicles are docked at the presynaptic plasma membrane) between control and LPS-injected mice (Table 1). We then examined the 2-μm circular area surrounding each neuronal soma to search for ‘orphaned’ inhibitory synapses. In total, we identified 141 and 121 inhibitory synapses from PBS- and LPS-injected mice, respectively, but failed to detect any presynaptic terminals detached from the neuronal surface, indicating that contact with activated microglia is required for LPS-induced synaptic stripping.

We also investigated whether excitatory synapses are affected by microglial activation following LPS treatment. To this end, we quantified the number of excitatory synapses in the neuropil in the motor cortex in both PBS- and LPS-injected mice. Excitatory synapses are identified by their prominent, thickened post-synaptic density and association with dendritic spines (Fig. 2d, arrows). There was no difference in the numbers of excitatory synapse between the two groups (Fig. 2e). Therefore, the LPS-induced synaptic reduction appears to be specific for inhibitory neurotransmission.

### Reduced inhibitory innervation increases neuronal synchronicity

Once inhibitory input is reduced, neurons become more excitable. For instance, temporary disruption of inhibitory synapses by the downregulation of GABA receptors can facilitate NMDA channel activity, which induces increased synchronization of neuronal firing^26^. We next asked if the observed microglial synaptic displacement modulates the firing of cortical neurons in a similar way. To address this question, we implanted microelectrode arrays into layers III/V of the motor cortex (Supplementary Fig. 2) of Sprague–Dawley (SD) rats and monitored the neuronal activity. Local field potentials were recorded with a wireless telemetric system, while the rats remained fully awake and freely moving. Due to the relatively large size of the wireless transmitter, which fits the head of a rat well but is cumbersome and impractical for a mouse, we chose to use rats for these experiments instead of mice. This is feasible because LPS-induced microglial activation in rats is identical to that in mice (Supplementary Fig. 3).

**Figure 3:**
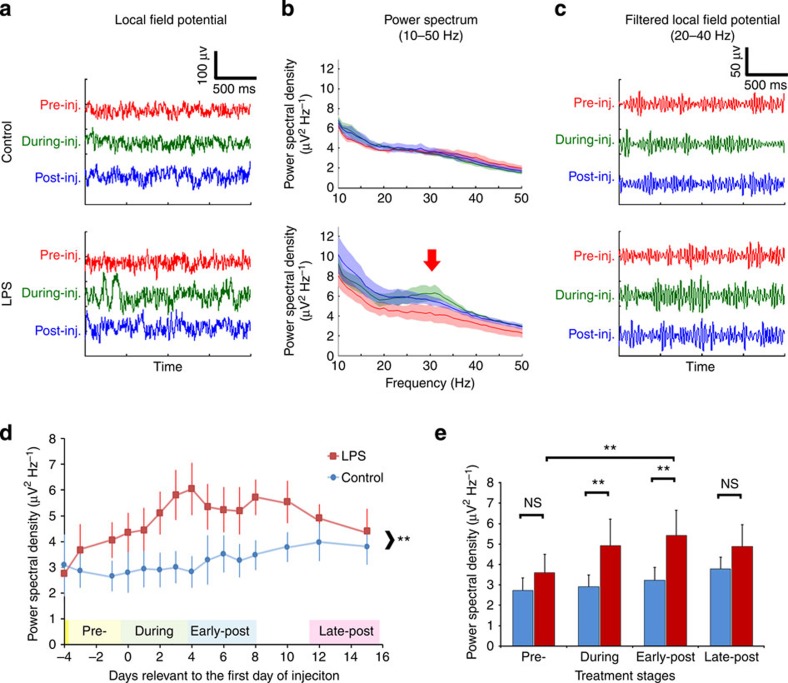
Reduced GABAergic neurotransmission results in increased neuronal firing. (**a**) Representative raw traces of local field potentials recorded from motor cortex of control (top) or LPS-injected animals (bottom) before (red), during (green) or after (blue) the 4-day injection regimen. (**b**) Power spectra density between 10 and 50-Hz frequencies of individual animals. Arrow indicates an increase in power spectra in the 20–40 Hz band in LPS-treated animals. Solid lines indicate mean power and shades represent s.d.; colour scheme is the same as in **a**. (**c**) Local field potentials were band filtered to show the power of the 20–40 Hz frequency. (**d**) Power spectral densities of low γ-band (20–40 Hz) field potentials were calculated for control (blue, *n*=3) and LPS-injected (red, *n*=5) animals. Data are represented as mean±s.e.m before (‘pre-’), during (‘0’ denotes first day of LPS injection) and after (‘early-’ or ‘late-post’) LPS injections. **P<0.01; two-way ANOVA. (**e**) The recordings were grouped into four stages relative to the LPS injections. The mean+s.e.m of each stage are shown. **P<0.01; NS, not significant; two-way ANOVA.

Local field potentials (Fig. 3a) of a representative animal from the LPS treatment group demonstrates a broadband increase in power across the frequency spectrum centred on the 20–40 Hz frequency band (referred to as γ-band) during and after administration of LPS (arrow, Fig. 3b). In contrast, a representative animal in the control group demonstrates a consistent power spectrum throughout the experiments (Fig. 3b). An increase in power spectral density is an indication of increased firing synchronicity of local neuronal ensembles. In order to make group comparisons between the LPS and control groups, we extracted the power from 20–40 Hz frequency band (Fig. 3c) and examined the aggregate power for each group relative to the treatment phases (Fig. 3d,e).

Animals treated with four daily injections of LPS had a significant increase in γ-band oscillatory activity when compared with PBS-injected controls (Fig. 3d). This increase peaked at 1 DPI and persisted for at least 5 days (Fig. 3d). Next, we grouped the time course of the treatment into four stages: (1) pretreatment, that is, before the initiation of LPS injections; (2) the time during the four daily LPS injections; (3) early post treatment, which includes the first 4 days after the final LPS injection; and (4) late post treatment, which includes days 7 to 12 after the final injection (Fig. 3d). Two-way analysis of variance (ANOVA) analysis of this ‘binned’ data indicates that there was a significant increase in γ-band oscillation in the ‘during’ and ‘early-post’ periods following LPS treatment compared with PBS controls (Fig. 3e). The increase in the ‘during’ phase is mostly attributable to the last 2 days of LPS injections, as the power spectral density remained low for the first 2 days (Fig. 3d). Furthermore, when compared longitudinally, LPS treatment significantly elevated neuronal synchronization in the ‘early-post’ stage compared with the ‘pre-’ stage in the same animals (Fig. 3e). This increased synchronicity gradually returned to control levels and the statistical differences between controls and the LPS-treated animals were not significant during the ‘late-post’ period (Fig. 3e). In contrast, the power of the γ-band activity in the control animals was relatively stable throughout the entire recording period (Fig. 3d,e). Interestingly, the progression of this neuronal oscillation concurs with the time course of microglial activation following LPS stimulation (Fig. 1), as both peaked in the early days immediately following four daily LPS injections.

### Neuronal synchronization increases CREB phosphorylation

**Figure 4:**
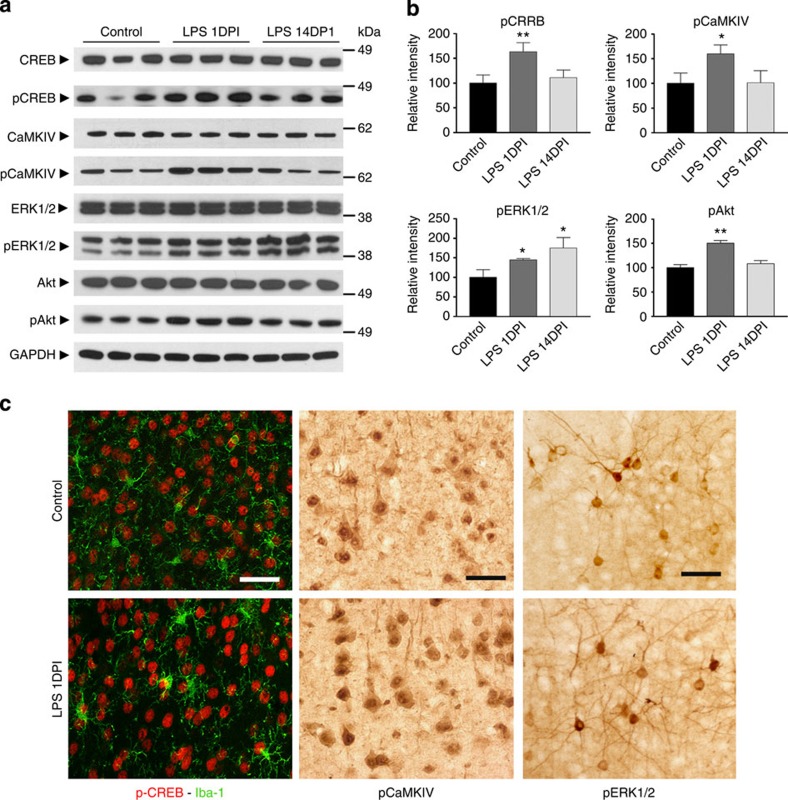
Increased neuronal synchronization is associated with increased phosphorylation of CREB. (**a**) Comparison of total and phosphorylated forms of CREB, CaMKIV, ERK1/2 and Akt in cortices from PBS-injected mice at 1DPI (control) and LPS-injected mice at 1 or 14 DPI. GAPDH was used as protein loading controls. Full western blot images are shown in Supplementary Fig. 6. (**b**) Densitometry quantification of **a** with the relative intensity of the controls set as 100%. (**c**) Immunohistochemistry demonstrates the enrichment of pCREB, CaMKIV and ERK1/2 in cortical neurons of LPS-injected mice at 1 DPI. Data are presented as mean+s.e.m. *P<0.05, **P<0.01; one-way ANOVA. Scale bar, 40 μm.

Increased neuronal synchronization is critical for synaptic NMDAR-mediated neuronal survival^27^. NMDAR activation results in increased cytosolic Ca^2+^ levels, which can subsequently activate Ca^2+^/calmodulin-dependent kinase IV (CaMKIV) and ERK1/2 (ref. 27). While levels of both CaMKIV and ERK1/2 were similar in control and LPS-treated animals, their activated (phosphorylated) forms were significantly increased in the cortex of LPS-treated mice compared with controls (Fig. 4a,b). It has been shown that both pCaMKIV and pERK1/2 can catalyse the activation of CREB^27^. While CREB levels were similar in the cortices of saline- and LPS-treated mice (Fig. 4a), phosphorylated CREB (pCREB) was significantly increased in the LPS-treated group (Fig. 4b). Activation of NMDARs can also mediate antiapoptotic effects through a mitogen-activated protein kinase-dependent phosphorylation of the threonine/serine kinase Akt at Ser^473^ (ref. 28). Akt levels were similar in cortices of LPS-treated and control mice. In contrast, the levels of pAkt at Ser^473^ were significantly increased 1 day after four LPS injections (Fig. 4a,b). Expression of these activated molecules is specifically found in neurons, as confirmed by pCREB, pCaMKIV and pERK1/2 immunohistochemistry at 1 DPI (Fig. 4c). Even though the subtle increase in intensity of these molecules can be appreciated in LPS-treated animals when compared with the controls, immunohistochemistry is not used to quantify the differences due to its intrinsic technical limitation but only to determine the cellular location of these molecules and to confirm that they are highly enriched in neurons.

### CREB activation induces expression of prosurvival molecules

**Figure 5:**
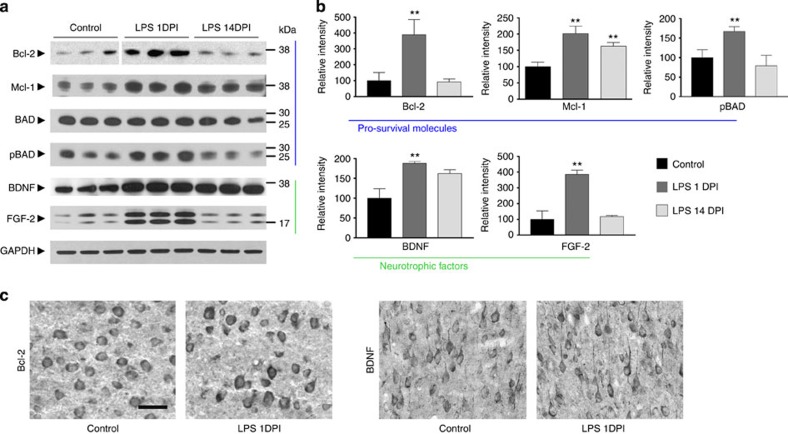
Neuronal expression of prosurvival genes is elevated in LPS-treated animals. (**a**) Comparison of antiapoptotic (Bcl-2, Mcl-1 and pBAD) and neurotrophic (BDNF and FGF-2) molecules in cortices from PBS-injected mice at 1 DPI and LPS-injected mice at 1 or 14 DPI by western blot analysis. Full western blot images are shown in Supplementary Fig. 7. (**b**) Densitometry quantification of **a** with the relative intensity of the controls set as 100%. There is a significant increase in the expression of antiapoptotic and neurotrophic molecules in LPS-injected animals at 1 DPI. (**c**) Immunohistochemistry demonstrates the enrichment of Bcl-2 and BDNF in cortical neurons of LPS-injected mice at 1 DPI. Data are presented as mean+s.e.m. **P<0.01; one-way ANOVA. Scale bar, 40 μm.

Phosphorylation of CREB is essential for neuronal survival during development or following injury through promoting the transcription of prosurvival molecules. Thus, we next quantified levels of proteins known to be regulated by activated pCREB and to confer neuroprotection. The expression of the antiapoptotic protein Bcl-2 was significantly increased (Fig. 5a,b). Myeloid cell leukaemia sequence (Mcl-1), a member of the Bcl-2 family that constitutes a negative regulator in the mitochondrial pathway leading to apoptosis, was also significantly increased (Fig. 5a,b). Levels of BAD (Bcl-associated death promoter), a Bcl-2/Bcl-X_L_-antagonist which is a proapoptotic member of the Bcl-2 family, were similar in control and LPS-treated animals. BAD phosphorylation at Ser^112^, however, was significantly increased in LPS-treated animals compared with saline-injected animals (Fig. 5a,b). Increase of BAD phosphorylation (and thus suppression of BAD activity) leads to the dissociation of BAD from Bcl-2 and thereby promotes cell survival^29^. The levels of brain-derived neurotrophic factor (BDNF), a neurotrophin known to be regulated by CREB^30^,^31^, were significantly increased in LPS-treated mice at 1 DPI. Fibroblast growth factor 2 (FGF-2), which can induce phosphorylation of BAD at Ser^112^ through the ERK1/2 signalling pathway^32^, was also significantly increased at 1 DPI (Fig. 5a,b). Bcl-2 and BDNF were highly enriched in neurons as shown by immunohistochemistry (Fig. 5c). Gene profiling of microglia isolated from PBS- and LPS-treated mice did not detect increased expression of antiapoptotic or neuroprotective transcripts following LPS treatment (Supplementary Fig. 4). Collectively, our data support the induction of antiapoptotic and prosurvival proteins in cortical neurons in LPS-treated mice at the apex of microglial activation.

### Suppression of microglial activation abolishes neuroprotection

**Figure 6:**
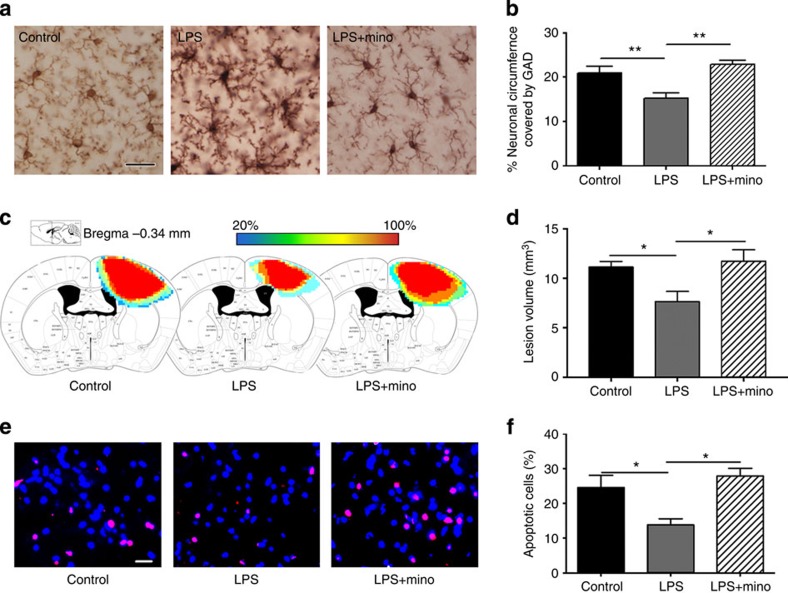
Suppression of microglial activation abolishes neuroprotection. (**a**) Cortical microglial morphology in mice injected with PBS, LPS or LPS+minocycline (LPS+mino). (**b**) Neuronal cell body circumferences covered by inhibitory presynaptic terminals were quantified as described in Fig. 1. (**c**–**f**) Mice treated with four daily injections of PBS, LPS or LPS+mino underwent cryogenic injury and were killed at 24 h post injury for TUNEL staining (**e**,**f**) or at 72 h post injury for lesion volume evaluation (**c**,**d**). (**c**) Tissue injury maps summarizing the average size of the brain lesion following cryo-injury for each group (Control, LPS, LPS+mino). (**d**) Quantification of lesion volumes. (**e**) Representative images of TUNEL^+^ apoptotic cells (red) in the penumbra of the cyro-lesions. Cellular nuclei are counterstained with 4',6-diamidino-2-phenylindole (blue). (**f**) Quantification of TUNEL^+^ cells. *N*=6–8 per group. Data are expressed as mean+s.e.m. *P<0.05, **P<0.01, one-way ANOVA. Scale bar, 20 μm in both **a** and **e**.

Multiple low-dose prophylactic LPS injections induce microglial activation and protect the brain against both cryogenic^8^ and fluid percussion injuries (Supplementary Fig. 5). To investigate whether microglial activation and synaptic stripping are essential components in this neuroprotection, we used the pharmacological inhibitor minocycline to suppress microglial activation. LPS-induced microglial activation and neuronal association were eliminated by the co-administration of minocycline (Fig. 6a). The neuronal cell surface occupied by inhibitory synapses was not reduced in mice treated with LPS and minocycline (Fig. 6b). Mice that were injected with PBS, LPS or LPS+minocycline were then subjected to cryogenic aseptic brain injury. Seventy-two hours after the injury, mice were killed and the sizes of their brain lesions were analysed (Fig. 6c,d). Mice that were pretreated with LPS had significantly smaller lesion volumes than the PBS control group, indicating that cortical tissue is protected against injury in the presence of activated microglia and synaptic displacement. However, this protection was abolished by the co-administration of LPS and minocycline (Fig. 6d). The decrease in lesion size in the LPS-treated animals relative to PBS control and LPS+minocycline group was associated with a decreased number of apoptotic cells found in the penumbra areas of the lesions 24 h after the injury (Fig. 6e,f), indicating that the reduced lesion size was associated with the induction of the antiapoptotic molecules following microglial synaptic displacement.

## Discussion

In this study, we demonstrate that the activated microglia mediate neuroprotection in the adult brain by removing inhibitory synapses from neurons and we identify increased neuronal expression of antiapoptotic and prosurvival molecules as the underlying mechanism of this neuroprotection. Specifically, we demonstrate that (1) activated microglia displace inhibitory synapses from neurons in the adult brain; (2) reduced inhibitory input increases cortical neuronal synchronization, resulting in increased Ca^2+^-mediated activation of antiapoptotic and prosurvival pathways; (3) pharmacological inhibition of microglial activation abolishes this neuroprotection; and (4) this neuroprotection is transient and the majority of cellular and molecular changes associated with this neuroprotection are reversible. We propose a neuroprotective function of activated microglia in the adult mammalian brain that may be operative in a variety of pathological CNS environments.

Recent studies have established that microglia prune synapses in an activity-dependent manner in healthy, developing brains^12^,^14^. Here, we extend the spectrum of microglial functions by presenting evidence that activated microglia participate in neuroprotection in the adult CNS via displacing inhibitory synapses. Microglia are activated following four daily intraperitoneal (IP) injections of LPS. These microglia alter their shape, redistribute their processes and physically associate with neuronal cell bodies. Triple-labelling of neuronal perikarya, microglia and GAD established that GAD-positive inhibitory presynaptic terminals are absent from neuronal surfaces that are ensheathed by microglia (Fig. 1). A critical question is whether microglia have a primary role in physically displacing presynaptic terminals or a secondary role of ensheathing the neuronal soma after presynaptic terminals withdraw. We addressed this question by investigating the 3D ultrastructure of microglia and presynaptic terminals on the neuronal surface at nanometre resolution. We found no evidence of presynaptic terminals withdrawing from the neuronal soma in the absence of microglial ensheathment (Table 1). In contrast, 3D-EM analysis detected microglial processes that physically separated presynaptic terminals from neuronal surface membranes (Fig. 2). These microglial processes tightly apposed both the presynaptic terminal and the post-synaptic neuronal soma. This evidence is consistent with active synaptic stripping by microglia because one would expect to observe a wider space between microglial contour and the retracted presynaptic terminal if the microglia were being recruited to ensheath the neurons following presynaptic terminal withdrawal. Regardless, future studies using multiphoton *in vivo* time-lapse imaging documenting the progression of microglial process insertion between the synaptic cleft would help address this question. 3D electron micrographs of a microglial process displacing only a portion of a presynaptic terminal provide compelling evidence for microglia-mediated synaptic stripping in the adult brain.

While many studies have focused on microglial pruning of excitatory synapses^11^,^12^,^14^, less is known about microglial modulation of inhibitory synapses. During development, microglial pruning of excitatory presynaptic terminals is associated with reduced electrical activity of the terminals^11^,^12^,^14^. It is therefore reasonable to suggest that activated microglia strip inhibitory synapses in response to reduced activity or altered function of the inhibitory synapses. GABA_A_ receptors are dynamically regulated and can be rapidly removed from the neuronal surface through receptor internalization^33^. It remains to be determined whether GABA_A_ receptor changes precede microglial-mediated synaptic displacement, and if so, whether activated microglia secrete factors that can contribute to its downregulation. In this regard, it was recently reported that the chemokine CXCL10 can decrease neuronal expression of GABAergic receptor subunits and reduce the firing of GABAergic synapses *in vitro*^34^. Microglia are a major source of CXCL10 following LPS treatment (Z. Chen, unpublished data). Therefore, it is possible that microglial production of CXCL10 reduces neuronal expression of GABAergic receptor subunits and inhibitory neurotransmission, which in turn attracts activated microglia to displace these weakened inhibitory synapses. Regardless of the precise neuronal mechanism that instigates activated microglia to strip inhibitory synapses, minocycline, an inhibitor of microglial activation, abolished microglial association with neurons and subsequent neuroprotection (Fig. 6). These data support microglial activation as an essential component of LPS-induced synaptic modulation and neuroprotection.

There are significant differences between microglia-mediated synaptic pruning during development and the synaptic stripping described in this report. Synaptic pruning in developing brain targets excitatory synapses and microglia engulf and destroy presynaptic terminals and post-synaptic dendritic spines^14^. Pruning of these synaptic components depends on the activation of microglia-specific phagocytosis mediated by the complement receptor 3/C3 pathway^14^. In the present model, activated microglia displace pre and post-synaptic components of inhibitory synapses but do not phagocytize presynaptic terminals or physically remove post-synaptic domains on the neuronal surface. Among all the microglia we examined in the triple-labelling images, there was no GAD^+^ punctae discernible within the microglial processes and cell bodies. In contrast to synaptic pruning during development, LPS-induced microglial synaptic modulation is not dependent on the complement cascade, as synaptic displacement was similar in WT and C1q KO mice (unpublished data). Microglial removal of synapses during development is permanent, while the microglia-mediated neuroprotective synaptic displacement described here is transient (Fig. 1). Therefore, the activation of the microglia-specific phagocytic complement pathway is unlikely to be involved in synaptic stripping, as destruction of synaptic components would be inefficient and delay reinnervation.

How do peripheral injections of LPS activate microglia and protect the brain? LPS is a surface antigen of Gram-negative bacteria that activates TLR4, a member of the Toll-like receptor family. Using reciprocal bone marrow transplantation protocols with wild-type and TLR4 knockout mice, we have previously established that LPS-induced microglial activation and neuroprotection requires TLR4 in the CNS but not in the peripheral immune system^8^. TLR4 expression in the brain is largely restricted to endothelial cells and microglia^35^. Since LPS does not cross the blood–brain barrier^36^, its mode of action in our model is via the activation of TLR4 on the luminal surface of brain blood vessels. Following IP injection, LPS enters the circulation and can activate endothelial TLR4 (ref. 37). The activation of endothelial TLR4 results in local cytokine secretion, which signals to the brain parenchyma and activates microglia. This stimulation has to be sustained for 3 or 4 days to achieve global microglial activation and subsequent neuroprotection, supporting the notion that successive signalling events occur, transitioning from brain endothelium to parenchymal microglia. This transient microglial activation and neuroprotection occurs in anticipation of bacterial invasion of the brain.

**Figure 7:**
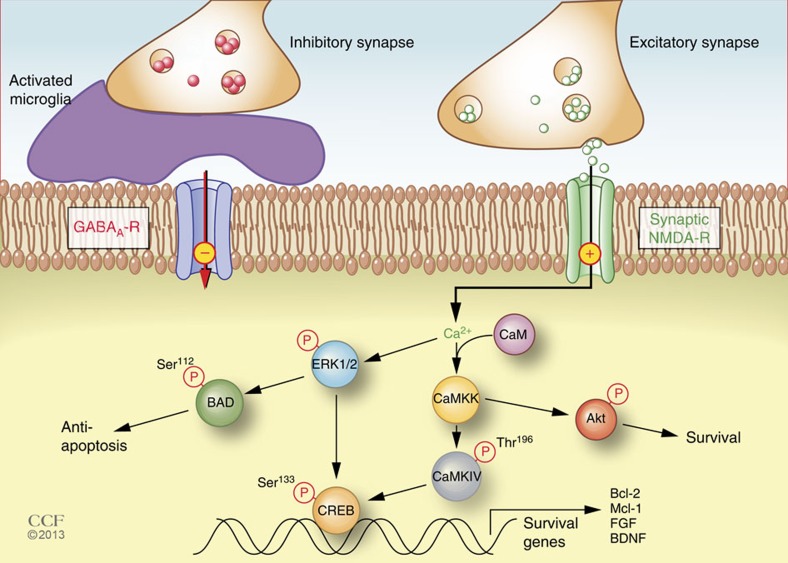
Model of activated microglia-mediated neuroprotection. Activated microglia displace presynaptic GABAergic terminals, which lowers the threshold for firing excitatory synaptic NMDARs. Increased activity of synaptic NMDARs (‘+’) elevates intracellular Ca^2+^ levels, which leads to activation of ERK1/2 and calmodulin-dependent phosphorylation of CaMKK and CaMKIV. Both activated ERK1/2 and CaMKIV can phosphorylate CREB at Ser^133^, which subsequently increases the transcription of neuroprotective genes and inhibitors of apoptosis. Activated ERK1/2 also phosphorylates BAD at Ser^112^, which dissociates from Bcl-2 and renders it active and consequently prevents apoptosis. In addition to CaMKIV, CaMKK can also phosphorylate Akt, which promotes cell survival through downregulation of pro-death signalling molecules. Thus, two molecular mechanisms are involved in the neuroprotection induced by microglial synaptic displacement: increased expression of survival and neurotrophic genes through CREB-mediated transcription and diminished apoptotic signals through phosphorylation of BAD and Akt.

How is microglia-mediated inhibitory synapse reduction neuroprotective? Reduction of inhibitory synaptic input is a documented neuroprotective mechanism due to the subsequent activation of synaptic NMDARs. Synaptic NMDAR activity is essential for neuronal survival and increased resistance to potential neural trauma^38^. The key to this neuroprotection is the influx of Ca^2+^ that accompanies synaptic NMDAR activation, which initiates intracellular Ca^2+^-dependent signalling events that culminate in *de novo* gene transcription or post-translational activation of antiapoptotic and neuroprotective molecules^27^,^39^. Pharmacological inhibition of GABAergic receptor activation by bicuculline lowers the threshold of excitatory synaptic NMDAR activity, resulting in increased synaptic NMDAR-dependent Ca^2+^ transients and neuroprotection^19^. Displacing of inhibitory presynaptic terminals by activated microglia mimics this bicuculline effect and induces neuroprotection by a similar mechanism. Essential aspects of the signalling cascades are summarized in Fig. 7.

Neurophysiological monitoring of cortical electrical activity in LPS-treated rodents detected a significant increase in neuronal spectral power in the γ-band (Fig. 3), strongly imitating the effects of bicuculline exerted on neural network synchronization. In agreement with the previous findings that bicuculline treatment results in NMDAR-dependent Ca^2+^ influx, we show here that phosphorylation of CaMKIV and ERK1/2, two Ca^2+^ transient-dependent kinases^40^, were increased in the cerebral cortex after LPS treatment (Fig. 4). Both kinases can mediate the phosphorylation of CREB at Ser^133^. Activated CREB promotes transcription of survival genes including BDNF, Bcl-2 and other inhibitors of apoptosis. In addition, ERK1/2 also post translationally deactivates the proapoptotic molecule BAD and phosphorylates Akt, which is known to promote cell survival via the downregulation of pro-death signalling molecules^38^. Reduced neuronal apoptosis and decreased brain lesion volume following LPS treatment establish that the increased expression of these antiapoptotic molecules and neurotrophic factors is associated with significant neuroprotection (Fig. 6).

It is important to stress that microglia-mediated reduction of GABAergic synapses increases the firing of synaptic NMDARs, which activates CREB and increases neuronal expression of antiapoptotic and neuroprotective molecules. In contrast, excessive activation of extrasynaptic NMDARs, which occurs following a stroke when very high levels of extracellular glutamate are released, deactivates CREB, decreases the expression of neuroprotective molecules and kills neurons via excitotoxicity^19^,^41^. This Yin and Yang of the consequences of synaptic versus extrasynaptic NMDAR activity has been described in detail^27^.

The physical association between activated microglia and neuronal perikarya has been described in a variety of disease states in the adult human brain, including neurosyphilis^5^, Alzheimer’s disease^42^, HIV dementia^43^, multiple sclerosis^44^ and ischaemic infarcts^45^,^46^. This microglial–neuronal interaction has often been interpreted as a mechanism by which activated microglia attack and destroy neurons. Most neurons ensheathed by activated microglia in the adult brain, however, appear healthy^5^,^44^. Therefore, we propose a sequence of activated microglia associations in the diseased brain. Initially, the activated microglia protect neurons by removal of inhibitory synapses. With time, however, this neuroprotection may fail and the neuron may instruct the activated microglia to transition from a neuroprotective to a phagocytic phenotype. The challenge, therefore, is to develop methods that distinguish the neuroprotective role of activated microglia from their phagocytic role of removing dying cells. By separating the neuroprotective and phagocytic phenotypes of activated microglia, the paradigm utilized in the present study provides an opportunity to identify a molecular signature for protective microglia. Future studies should establish the molecular phenotype of these protective microglia, determine if these cell are present in diseased brains and whether neurons ensheathed by activated microglia in diseased brain have reduced inhibitory innervation.

In conclusion, our data identify a neuroprotective role for activated microglia in the adult brain. We provide evidence that activated microglia strip inhibitory synapses from neurons and identify neuronal signalling pathways that protect neurons by increasing the expression of antiapoptotic and neuroprotective molecules. Collectively, these data raise the possibility that the common physical association between microglia and neurons in diseased brains reflect the neuroprotective role of the innate immune system.

## Methods

### Animals

Adult male C57BL/6J mice at 8–12 weeks of age were purchased from Jackson Labs; male SD rats (250–300 g body weight) were purchased from Harlan Laboratories. The animals were housed under pathogen-free conditions in a temperature- and humidity-controlled environment and given access to food and water *ad libitum*. Animals were allowed to acclimate to the environment for at least 5 days before being used for experiments. All experimental procedures were approved by the Institutional Animal Care and Use Committee (IACUC) of the Cleveland Clinic.

### LPS and minocycline treatment

Animals (mice or rats) received four intraperitoneal injections of LPS (Sigma *E. coli, serotype 055:B5*) in PBS over four consecutive days at a dose of 1.0 mg per kg. Control animals received similar injections of PBS. At 1, 7 or 14 days post injection (DPI), mice were anaesthetized and killed for brain tissue analysis as described below. In a separate group of mice, minocycline (50 mg kg^−1^) was injected (IP) for 4 days starting on the same day as the initial LPS treatment; minocycline and LPS injections were spaced 4 h apart. One day after the final injections, mice were either killed for brain tissue analysis or subjected to TBI as described below.

### Immunohistochemistry

LPS-, PBS- or minocycline-treated animals (*n*=3 per group) were anaesthetized and transcardially perfused with freshly prepared 4% paraformaldehyde in 0.08 M phosphate buffer (pH=7.4). Brains were removed, post-fixed overnight, cryoprotected in 20% glycerol and sectioned on a freezing sliding microtome. Free-floating coronal brain sections (30 μm) were pretreated with 3% H_2_O_2,_ 1% Triton X-100 in PBS and blocked with 3% normal goat serum. Sections were incubated in primary antibodies (Iba-1, 1:500, generated at the Cleveland Clinic Hybridoma Core; F4/80, 1:250, AbDSerotech; pERK1/2, 1:500, Cell Signaling; pCaMKIV, 1:200, Santa Cruz; Bcl-2, 1:200 and BDNF, 1:500, Abcam) overnight and labelled with the avidin–biotin complex (Vector Laboratories) and visualized with diaminobenzidine (DAB; Sigma). The DAB reaction product was enhanced with 0.04% osmium tetroxide (Electron Microscopy Sciences) for 30 s. Sections were mounted on glass slides in glycerol and cover-slipped for microscopic analysis. Digital images were photographed using a Leica DMR microscope fitted with an Optronics Magna Fire CCD colour video camera.

### Immunofluorescence and confocal microscopy

Confocal microscopy was performed as previously described^8^. Briefly, free-floating coronal brain sections (30 μm) were pretreated with 1% Triton X-100 in PBS and blocked with 3% normal goat serum. Sections were incubated with primary antibodies (mouse-anti-Iba-1, 1:500, CCF Hybridoma Core; rabbit-anti-GAD 65/67, 1:300, Millipore; goat anti-pCREB, 1:250, Santa Cruz) overnight at 4 °C and then stained with appropriate Alexa-488 or -647 conjugated secondary antibodies (Vector Laboratories) for 2 h at room temperature (RT). Neurons were subsequently stained using Neuro Trace Nissl stain (1:300 for 30 min at RT, Life Technologies). Sections were rinsed, mounted with vectashield (Vector Laboratories) and examined on a Leica TCS confocal microscope (Leica Microsystems). The percentage of microglia in contact with neurons was calculated by dividing the number of microglia associated with neurons over the total number of microglia in a micrograph field. The percentage of neuronal circumference covered by inhibitory synapses was calculated by dividing the length of GAD 65/67 punctae around the neuronal soma by neuronal circumference.

### 3D-EM

3D-EM was performed in a Carl Zeiss Sigma VP scanning electron microscope containing a 3View in-chamber ultramicrotome system and Gatan high sensitivity, low-kV backscattered electron detector (Gatan, England). Tissue preparation and imaging were performed as previously described^23^. Briefly, mice were perfused at 1 day post LPS or PBS injections with cacodylate-buffered 2.5% glutaraldehyde and 4% paraformaldehyde. Brains were removed and 1-mm coronal slices were cut with a mouse brain matrix (Electron Microscopy Sciences). A 1 mm^3^ tissue block was further dissected from the motor cortex and treated with 2% OsO_4_-ferricyanide, followed sequentially by thiocarbohydrazide, aqueous OsO_4_ (2%), aqueous uranyl acetate and Walton’s lead aspartate stain^47^, before they were embedded in Epon resin (all chemicals from Electron Microscopy Services). Samples were trimmed to include projection neurons in cortical layers III and IV. Serial images of the block face were generated by automated repeated cycles of cutting and scanning. Images were acquired with a 2.25 kV beam in high vacuum mode. Sets of 250–500 images at 75 nm steps (that is, section thickness) were obtained at 8–10 nm per pixel resolution, producing images of 78 × 78 μm. Images were registered, corrected for histogram contrast and derivative stacks generated using ImageJ software with FIJI plug-in sets. 3D reconstructions were generated by tracing cell profiles using Reconstruct software^48^ and meshes exported to Blender software ( www.blender.org) for 3D rendering.

### Electrophysiology recording in free-roaming awake animals

Male SD rats (250–300 g, *n*=3 for control group and *n*=5 for LPS group) were anaesthetized and mounted in prone position on a stereotaxic frame (Kopf Instruments). An incision was made in the scalp to expose the skull. Craniotomy was performed to remove a 1 × 2 mm piece of the skull, at −2.0 mm anterior and 2.0 mm lateral to bregma over the right parietal cortex. A custom-made microelectrode array (Alpha Omega) with five channels (50 μm in diameter and 50 μm apart, resistance 0.4–0.6 MΩ) was implanted into layers III/V of the motor cortex. The electrode array was connected to a cranial mount, which allows for transmission of the electrophysiological signals via a wireless transmitter. This telemetric system offers significant advantages in that it does not tether the animal to the recording system, hence the animal is able to ambulate freely. The animals were allowed to recover for a week after the implantation. Thereafter, they were monitored on a daily basis for 60 min per day and randomly assigned to either control or LPS treatment group. The measured local field potential was processed with a band-passing filter of 1.2–300 Hz, amplified ( × 1,020) and digitized (787.8 samples per second) with 12 bits resolution. The daily recordings were maintained for 3 weeks. Electrophysiology data were analysed offline with in-house built programme using MatLab software (Math Works). Signals whose amplitudes are greater than 500 μV or five times the s.d. were considered artifacts and were excluded from the analysis. For each recorded signal per channel, the low γ-band (20–40 Hz) power spectral density per each second was calculated with a Hamming window of 2 s.

### Protein isolation and western blotting

LPS- or PBS-treated animals (*n*=3 per group) were decapitated and their cortices were removed, frozen in liquid nitrogen and stored at −80 °C. The samples were homogenized in ice-cold lysis buffer containing 20 mM HEPES (pH 7.9), 400 mM KCl, 5 mM MgCl_2_, 1 mM EDTA, 1 mM EGTA, 300 mM sucrose, 1 mM of phenylmethylsulphonyl fluoride, 5 μg ml^−1^ protease inhibitor cocktail (Sigma) and 5 μg ml phosphatase inhibitor cocktail (Pierce). The homogenates were centrifuged at 13,000 r.p.m. for 15 min at 4 °C and the supernatants were stored at −80 °C until use. Protein concentrations were determined using the Bradford method (BioRad) per manufacturer’s instructions. Samples were separated by SDS-polyacrylamide gel electrophoresis performed with the XCell SureLock system (Invitrogen), using precast gradient gels (4–12%). Thirty micrograms of total protein was loaded in each lane. The gels were transferred to polyvinylidene difluoride membranes (Millipore), which were then blocked for 1 h at RT with 10% (w/v) nonfat-dried milk (BioRad) in tris-buffered saline with 0.1% Tween-20. The membranes were placed in primary antibodies overnight at 4 °C, incubated in horseradish peroxidase-tagged secondary antibodies for 1 h at RT, treated with Supersignal Pico detection reagent (Pierce) and exposed to Kodak X-Omat film (Kodak). Membranes were then stripped and subsequently reprobed with glyceraldehyde-3-phosphate dehydrogenase (GAPDH) antibody (Millipore). Films were scanned and the average intensity was quantified with FIJI with controls set arbitrarily to 100. Primary antibodies used in these studies are well characterized and include CREB (1:2,000), Akt (1:1,000), pAkt (1:1,000), CaMKIV (1:1,000), pERK1/2 (1:700), Bad (1:1,000), pBad (1:1,000, all from Cell Signaling Technology); pCamKIV (1:200, Santa Cruz); BDNF (1:500) and Bcl-2 (1:500, Abcam); Mcl-1 (1:10,000, BioLegend); pCREB (1:500), ERK1/2 (1:1,000), FGF-2 (1:500) and GAPDH (1:30,000, Millipore).

### Mouse models of TBI and quantification of lesion volume

Two TBI models (cryogenic and fluid percussive) were used in our studies. The cryogenic injury model has been used by our laboratory previously^8^. Briefly, mice (*n*=6–8 per group) were anaesthetized with IP injections of Ketamine (100 mg kg^−1^) and Xylazine (10 mg kg^−1^). The surgeon was blinded to the treatments (PBS, LPS or LPS+minocycline) given to the mice. A 10-mm midline sagittal incision was made in the scalp. A unilateral aseptic cryogenic injury of the right parietal cortex was induced by applying a 3.5-mm diameter metallic probe, which was precooled in liquid nitrogen, on the skull for 15 s, resulting in blanching of the skull surface. The skin incision was closed and mice were allowed to recover. To create fluid percussive injuries, a 3.5 mm diameter craniotomy was performed over the motor cortex. A custom-made Luer lock hub was attached to the skull surrounding the craniotomy. A controlled hydraulic impact of 1 atm (14.7 psi) was delivered to the exposed brain through the hub using a percussion device (Dragonfly R&D Inc.). The hub was then removed, the skin was closed, and the mice were allowed to recover. To quantify lesion volumes, 72 h after injury, mice were deeply anaesthetized and transcardially perfused with 4% paraformaldehyde and brains were removed for assessment of brain injury. Serial coronal brain sections (30 μm) were cut on a cryostat (Leica) and collected every 300 μm throughout the entire brain and stained with Geimsa stain (Sigma). Microscope slides were then scanned on a slide scanner (Microtek) and analysed with in-house developed software as previously described^49^. Lesion volumes were calculated by multiplying the total lesion area by the section interval thickness and expressed in mm^3^.

### TUNEL assay

Terminal deoxynucleotidyl transferase mediated dUTP Nick End Labelling (TUNEL) assays were used for quantification of apoptotic cells. Mice were perfused 24 h following injury and their brains were processed as described above. Apoptotic cells characterized by DNA fragmentation were detected using a Klenow-FragEL DNA fragmentation detection kit (Calbiochem) and the nuclei were counter stained with 4′,6-diamidino-2-phenylindole. Two slides from each brain taken from the center of the lesion were examined. Cell counting was performed in the penumbra of the lesion defined by a 150-μm thick area at the deepest border of the lesion. TUNEL^+^ cells were counted in at least three mice per group.

### Microglia isolation and RNA microarray analysis

Mice were injected with LPS or saline as described above (*n*=6 per group). Twenty-four hours after the final IP injection, mice were anaesthetized and transcardially perfused with PBS. Brains were rapidly removed, minced and trypsinized (0.25%) for 60 min at 37 °C. Trypsin was quenched by adding 20% fetal bovine serum in Dulbecco’s minimum essential medium. The cell suspension was separated on a discontinuous 30/70% percoll gradient (GE Healthcare). Myelin and cell debris were discarded from the top of the 30% percoll layer and the cell enriched fraction at the top of the 70% percoll was collected. The cells were incubated with fluorescently conjugated CD11b and CD45 antibodies (1:250, BD Pharmingen) at 4 °C for 30 min. Fluorescently labelled cell populations were separated using a Becton-Dickinson FACSAria I Flow Sorter. Purity of the isolated microglia (CD11b-positive/CD45 intermediate) is routinely over 90%. Total RNAs were then isolated using Trizol reagent (Invitrogen), and biotinylated cRNA probes were generated and hybridized to 430A mouse arrays (Affymetrix). Following hybridization with biotinylated probes, arrays were washed, stained and scanned using the Gene Array scanner (Affymetrix). Scanned images were inspected and quality control procedures standardized in the laboratory and recommended by the manufacturer were utilized to eliminate outliers. Initial data analysis was performed using Microarray Analysis Suite software 5.0 (MAS 5.0, Affymetrix). Resultant data files were used to compile the gene profiles of LPS-treated microglia for the levels of prosurvival molecules.

### Statistical analysis

Prism for Windows (GraphPad Software) was used for unpaired Student’s *t*-test or one-way ANOVA analysis where appropriate. If the ANOVA produced a significant result, the Bonferroni method was applied for multiple comparisons among the individual groups. Electrophysiological data were initially processed with an in-house developed programme on the MatLab platform (MathWorks), followed by unbalanced two-way ANOVA analysis with Bonferroni *post hoc* tests using SPSS software (IBM). Results are presented as mean±s.d. or otherwise stated. Statistical significance was defined as *P*<0.05.

## Author contributions

B.D.T. conceived and supervised the project. Z.C., W.J., J.T.G. and B.D.T. designed the study. Z.C., W.J., W.H., Z.C.G. and R.D. performed the experiments. Z.C., W.J., G.J.K., H.-J.P., J.T.G., W.H., J.C., R.D. and B.D.T. analysed the data. Z.C., W.J., J.T.G. and B.D.T. wrote the manuscript.

## Additional information

**How to cite this article:** Chen, Z. *et al*. Microglial displacement of inhibitory synapses provides neuroprotection in the adult brain. *Nat. Commun.* 5:4486 doi: 10.1038/ncomms5486 (2014).

## Supplementary Material

Supplementary Information Supplementary Figures 1-7

## Figures and Tables

**Table 1:** Synaptic coverage on neurons without ensheathing microglia.

**Treatment**	**Neuronal diameter**		**Number of synapses per neuron**	**Average length of active zones (μm)**
	**Major axis**	**Minor axis**		
PBS (*n*=15)	13.45±2.37	10.55±2.28	5.53±1.13	0.96±0.17
LPS (*n*=15)	13.02±2.11	10.15±1.19	5.73±0.80	1.02±0.20
*P*-value	0.607	0.554	0.579	0.393

LPS, lipopolysaccharide.

Mean±s.d.; *t*-test.

Major axis: the height of the pyramidal cell profile, which approximates a prolate spheroid; minor axis: the width of the pyramidal cell profile.

## References

[b1] Ransohoff R. M. & Perry V. H. Microglial physiology: unique stimuli, specialized responses. Annu. Rev. Immunol. 27, 119–145 (2009). 19302036 10.1146/annurev.immunol.021908.132528

[b2] Paolicelli R. C. . Synaptic pruning by microglia is necessary for normal brain development. Science 333, 1456–1458 (2011). 21778362 10.1126/science.1202529

[b3] Li Y., Du X. F., Liu C. S., Wen Z. L. & Du J. L. Reciprocal regulation between resting microglial dynamics and neuronal activity in vivo. Dev. Cell 23, 1189–1202 (2012). 23201120 10.1016/j.devcel.2012.10.027

[b4] Aguzzi A., Barres B. A. & Bennett M. L. Microglia: scapegoat, saboteur, or something else? Science 339, 156–161 (2013). 23307732 10.1126/science.1227901 PMC4431634

[b5] Graeber M. B. Changing face of microglia. Science 330, 783–788 (2010). 21051630 10.1126/science.1190929

[b6] Biber K., Owens T. & Boddeke E. What is microglia neurotoxicity (not)? Glia 62, 841–854 (2014). 24590682 10.1002/glia.22654

[b7] Kerschensteiner M., Stadelmann C., Dechant G., Wekerle H. & Hohlfeld R. Neurotrophic cross-talk between the nervous and immune systems: implications for neurological diseases. Ann. Neurol. 53, 292–304 (2003). 12601697 10.1002/ana.10446

[b8] Chen Z. . Lipopolysaccharide-induced microglial activation and neuroprotection against experimental brain injury is independent of hematogenous TLR4. J. Neurosci. 32, 11706–11715 (2012). 22915113 10.1523/JNEUROSCI.0730-12.2012 PMC4461442

[b9] Hellwig S., Heinrich A. & Biber K. The brain’s best friend: microglial neurotoxicity revisited. Front Cell Neurosci. 7, 71 (2013). 23734099 10.3389/fncel.2013.00071 PMC3655268

[b10] Nimmerjahn A., Kirchhoff F. & Helmchen F. Resting microglial cells are highly dynamic surveillants of brain parenchyma in vivo. Science 308, 1314–1318 (2005). 15831717 10.1126/science.1110647

[b11] Wake H., Moorhouse A. J., Jinno S., Kohsaka S. & Nabekura J. Resting microglia directly monitor the functional state of synapses in vivo and determine the fate of ischemic terminals. J. Neurosci. 29, 3974–3980 (2009). 19339593 10.1523/JNEUROSCI.4363-08.2009 PMC6665392

[b12] Tremblay M. E., Lowery R. L. & Majewska A. K. Microglial interactions with synapses are modulated by visual experience. PLoS Biol. 8, e1000527 (2010). 21072242 10.1371/journal.pbio.1000527 PMC2970556

[b13] Stevens B. . The classical complement cascade mediates CNS synapse elimination. Cell 131, 1164–1178 (2007). 18083105 10.1016/j.cell.2007.10.036

[b14] Schafer D. P. . Microglia sculpt postnatal neural circuits in an activity and complement-dependent manner. Neuron 74, 691–705 (2012). 22632727 10.1016/j.neuron.2012.03.026 PMC3528177

[b15] Blinzinger K. & Kreutzberg G. Displacement of synaptic terminals from regenerating motor neurons by microglial cells. Z Zellforsch. Mikrosk. Anat. 85, 145–157 (1968). 5706753 10.1007/BF00325030

[b16] Kreutzberg G. W. Microglia: a sensor for pathological events in the CNS. Trends Neurosci. 19, 312–318 (1996). 8843599 10.1016/0166-2236(96)10049-7

[b17] Ikonomidou C. . Blockade of NMDA receptors and apoptotic neurodegeneration in the developing brain. Science 283, 70–74 (1999). 9872743 10.1126/science.283.5398.70

[b18] Hardingham G. E., Arnold F. J. & Bading H. Nuclear calcium signaling controls CREB-mediated gene expression triggered by synaptic activity. Nat. Neurosci. 4, 261–267 (2001). 11224542 10.1038/85109

[b19] Hardingham G. E., Fukunaga Y. & Bading H. Extrasynaptic NMDARs oppose synaptic NMDARs by triggering CREB shut-off and cell death pathways. Nat. Neurosci. 5, 405–414 (2002). 11953750 10.1038/nn835

[b20] Monti B., Marri L. & Contestabile A. NMDA receptor-dependent CREB activation in survival of cerebellar granule cells during in vivo and in vitro development. Eur. J. Neurosci. 16, 1490–1498 (2002). 12405962 10.1046/j.1460-9568.2002.02232.x

[b21] Peters A. & Harriman K. M. Different kinds of axon terminals forming symmetric synapses with the cell bodies and initial axon segments of layer II/III pyramidal cells. III. Origins and frequency of occurrence of the terminals. J. Neurocytol. 21, 679–692 (1992). 1403013 10.1007/BF01191729

[b22] Denk W. & Horstmann H. Serial block-face scanning electron microscopy to reconstruct three-dimensional tissue nanostructure. PLoS Biol. 2, e329 (2004). 15514700 10.1371/journal.pbio.0020329 PMC524270

[b23] Ohno N. . Myelination and axonal electrical activity modulate the distribution and motility of mitochondria at CNS nodes of Ranvier. J. Neurosci. 31, 7249–7258 (2011). 21593309 10.1523/JNEUROSCI.0095-11.2011 PMC3139464

[b24] Peters A., Palay S. L. & Webster H. d. The Fine Structure of The Nervous System: Neurons and Their Supporting Cells Oxford University Press (1991).

[b25] Gray E. G. Axo-somatic and axo-dendritic synapses of the cerebral cortex: an electron microscope study. J. Anat. 93, 420–433 (1959). 13829103 PMC1244535

[b26] Woo N. H. & Lu B. Regulation of cortical interneurons by neurotrophins: from development to cognitive disorders. Neuroscientist 12, 43–56 (2006). 16394192 10.1177/1073858405284360

[b27] Hardingham G. E. & Bading H. The Yin and Yang of NMDA receptor signalling. Trends Neurosci. 26, 81–89 (2003). 12536131 10.1016/S0166-2236(02)00040-1

[b28] Lafon-Cazal M., Perez V., Bockaert J. & Marin P. Akt mediates the anti-apoptotic effect of NMDA but not that induced by potassium depolarization in cultured cerebellar granule cells. Eur. J. Neurosci. 16, 575–583 (2002). 12270033 10.1046/j.1460-9568.2002.02124.x

[b29] Zha J., Harada H., Yang E., Jockel J. & Korsmeyer S. J. Serine phosphorylation of death agonist BAD in response to survival factor results in binding to 14-3-3 not BCL-X(L). Cell 87, 619–628 (1996). 8929531 10.1016/s0092-8674(00)81382-3

[b30] Tao X., Finkbeiner S., Arnold D. B., Shaywitz A. J. & Greenberg M. E. Ca2+ influx regulates BDNF transcription by a CREB family transcription factor-dependent mechanism. Neuron 20, 709–726 (1998). 9581763 10.1016/s0896-6273(00)81010-7

[b31] Shieh P. B., Hu S. C., Bobb K., Timmusk T. & Ghosh A. Identification of a signaling pathway involved in calcium regulation of BDNF expression. Neuron 20, 727–740 (1998). 9581764 10.1016/s0896-6273(00)81011-9

[b32] Hsuan S. L., Klintworth H. M. & Xia Z. Basic fibroblast growth factor protects against rotenone-induced dopaminergic cell death through activation of extracellular signal-regulated kinases 1/2 and phosphatidylinositol-3 kinase pathways. J. Neurosci. 26, 4481–4491 (2006). 16641227 10.1523/JNEUROSCI.4922-05.2006 PMC6674070

[b33] Moss S. J. & Smart T. G. Constructing inhibitory synapses. Nat. Rev. Neurosci. 2, 240–250 (2001). 11283747 10.1038/35067500

[b34] Cho J., Nelson T. E., Bajova H. & Gruol D. L. Chronic CXCL10 alters neuronal properties in rat hippocampal culture. J. Neuroimmunol. 207, 92–100 (2009). 19167097 10.1016/j.jneuroim.2008.12.007 PMC2817940

[b35] Chakravarty S. & Herkenham M. Toll-like receptor 4 on nonhematopoietic cells sustains CNS inflammation during endotoxemia, independent of systemic cytokines. J. Neurosci. 25, 1788–1796 (2005). 15716415 10.1523/JNEUROSCI.4268-04.2005 PMC6725921

[b36] Coceani F., Lees J. & Dinarello C. A. Occurrence of interleukin-1 in cerebrospinal fluid of the conscious cat. Brain Res. 446, 245–250 (1988). 3259449 10.1016/0006-8993(88)90883-9

[b37] Hirano S. Migratory responses of PMN after intraperitoneal and intratracheal administration of lipopolysaccharide. Am. J. Physiol. 270, L836–L845 (1996). 8967519 10.1152/ajplung.1996.270.5.L836

[b38] Papadia S. & Hardingham G. E. The dichotomy of NMDA receptor signaling. Neuroscientist 13, 572–579 (2007). 18000068 10.1177/10738584070130060401 PMC2830536

[b39] Martin H. G. & Wang Y. T. Blocking the deadly effects of the NMDA receptor in stroke. Cell 140, 174–176 (2010). 20141829 10.1016/j.cell.2010.01.014

[b40] Hansen H. H. . Mechanisms leading to disseminated apoptosis following NMDA receptor blockade in the developing rat brain. Neurobiol. Dis. 16, 440–453 (2004). 15193300 10.1016/j.nbd.2004.03.013

[b41] Tu W. . DAPK1 interaction with NMDA receptor NR2B subunits mediates brain damage in stroke. Cell 140, 222–234 (2010). 20141836 10.1016/j.cell.2009.12.055 PMC2820131

[b42] Haga S., Akai K. & Ishii T. Demonstration of microglial cells in and around senile (neuritic) plaques in the Alzheimer brain. An immunohistochemical study using a novel monoclonal antibody. Acta Neuropathol. 77, 569–575 (1989). 2750476 10.1007/BF00687883

[b43] Yoshioka M. . Simultaneous detection of ferritin and HIV-1 in reactive microglia. Acta Neuropathol. 84, 297–306 (1992). 1414282 10.1007/BF00227823

[b44] Peterson J. W., Bo L., Mork S., Chang A. & Trapp B. D. Transected neurites, apoptotic neurons, and reduced inflammation in cortical multiple sclerosis lesions. Ann. Neurol. 50, 389–400 (2001). 11558796 10.1002/ana.1123

[b45] Gehrmann J., Bonnekoh P., Miyazawa T., Hossmann K. A. & Kreutzberg G. W. Immunocytochemical study of an early microglial activation in ischemia. J. Cereb. Blood Flow Metab. 12, 257–269 (1992). 1548298 10.1038/jcbfm.1992.36

[b46] Neumann J. . Microglia provide neuroprotection after ischemia. FASEB J. 20, 714–716 (2006). 16473887 10.1096/fj.05-4882fje

[b47] Walton J. Lead asparate, an en bloc contrast stain particularly useful for ultrastructural enzymology. J. Histochem. Cytochem. 27, 1337–1342 (1979). 512319 10.1177/27.10.512319

[b48] Fiala J. C. Reconstruct: a free editor for serial section microscopy. J. Microsc. 218, 52–61 (2005). 15817063 10.1111/j.1365-2818.2005.01466.x

[b49] Park H. J. . Semi-automated method for estimating lesion volumes. J. Neurosci. Methods 213, 76–83 (2013). 23261655 10.1016/j.jneumeth.2012.12.010 PMC3570743

