# Supplementary material for: Microglial displacement of inhibitory synapses provides neuroprotection in the adult brain
Source: Nat Commun. 2014 Jul 22;5:4486. doi: 10.1038/ncomms5486 (PMC4109015; doi:10.1038/ncomms5486)
Supplement: Supplementary Information — Supplementary Figures 1-7 [file ncomms5486-s1.pdf]

**Supplementary Information**

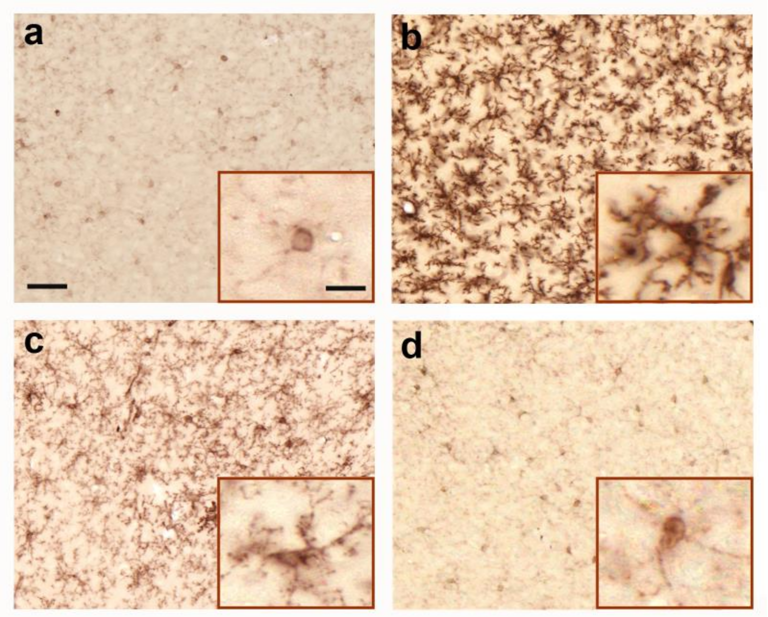


**Supplementary Figure 1 | Microglia are transiently activated by 4 daily LPS IP injections.**

Brain sections are stained with F4/80, a microglial activation marker, with individual microglia shown in insets. (**a**) Microglia in PBS treated control mice. (**b**) 24 hours after the final LPS treatment. (**c**) 7 days after the final LPS injection. (**d**) 14 days after the LPS-treatment. Bar= 60 μm in main panel or 10 μm in inset.

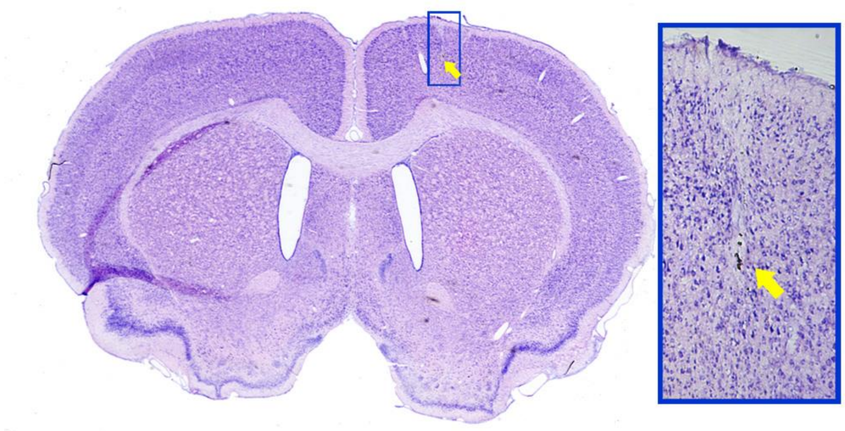

**Supplementary Figure 2 | Microelectrodes were placed in layer III/V of rat motor cortex.**

The tracts generated by implanted microelectrodes terminated in cortical layers III/V (arrow, inset) as confirmed by Giemsa staining.

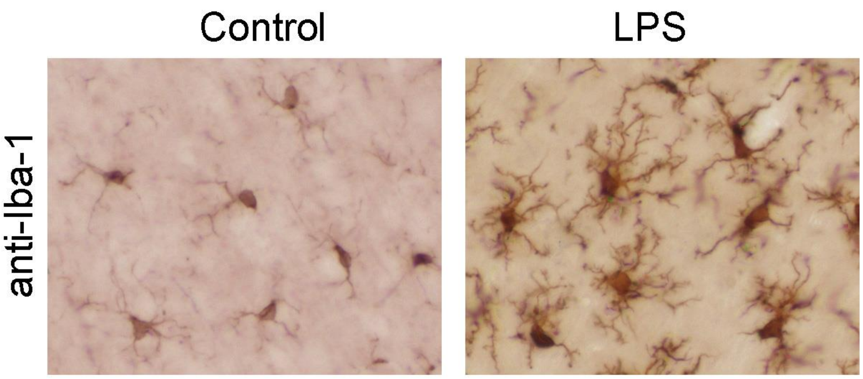


**Supplementary Figure 3 | Microglia in rat brain are activated by 4 daily IP injections of LPS.**

Four daily injections of LPS into the peritoneal cavity of rats activate microglia in an identical manner as to that described for mice (see Fig. 1a-c).

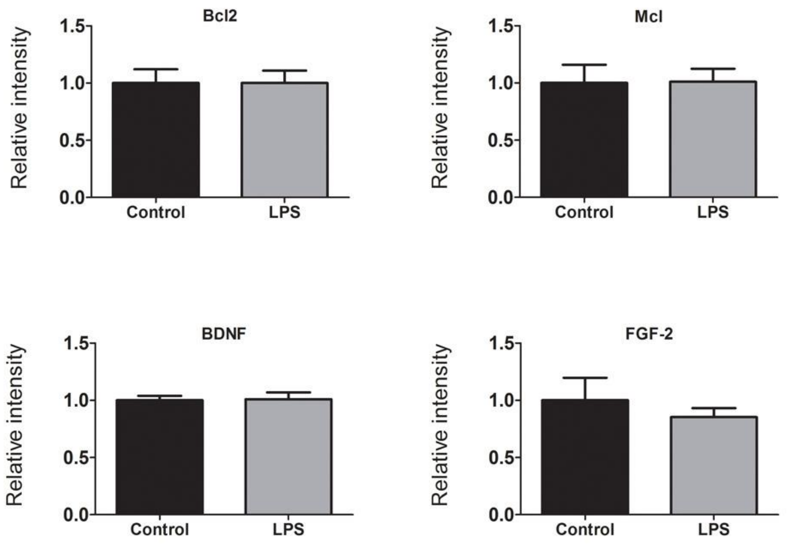


**Supplementary Figure 4 | Pro-survival molecules are not increased in purified microglia.** Microglia are purified by FACS from either PBS- or LPS-injected mice and their mRNA are isolated for microarray analysis. Data are shown as fold-increase compared to controls, which is set at 1.0.

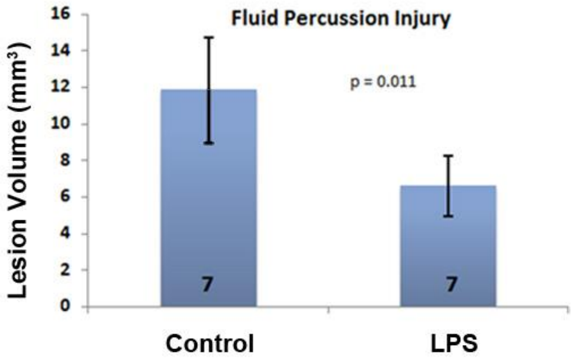


**Supplementary Figure 5 | LPS treatment reduces lesion size in a fluid percussive brain injury model.**

Mice (N=7 per group) were subjected to fluid percussion injury 24 hours after 4 daily injections of LPS or PBS, and their brain lesion volumes were analyzed 72 hours later.

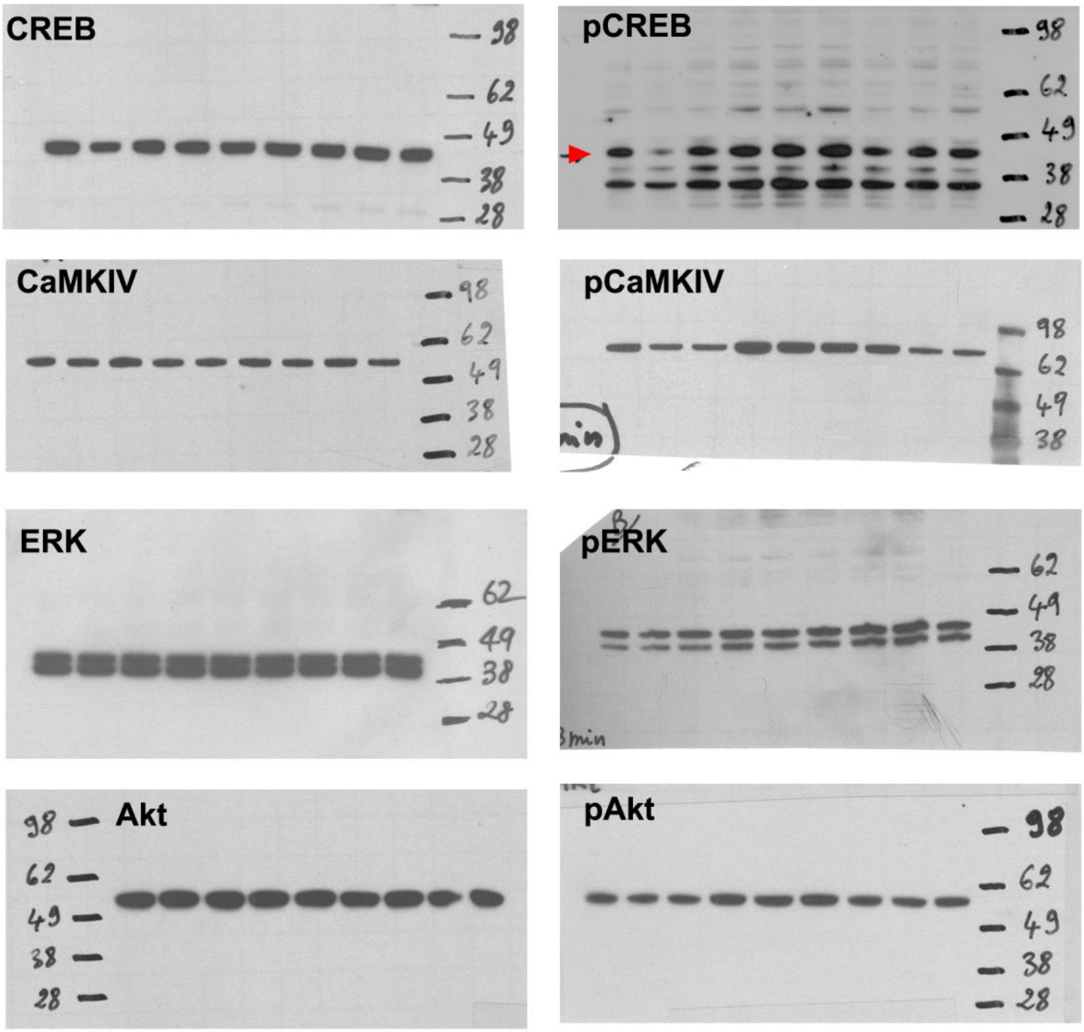


**Supplementary Figure 6 | Full western blots containing bands presented in Figure 4a.**

Arrowhead indicates band of interest if multiple bands appear in a lane.

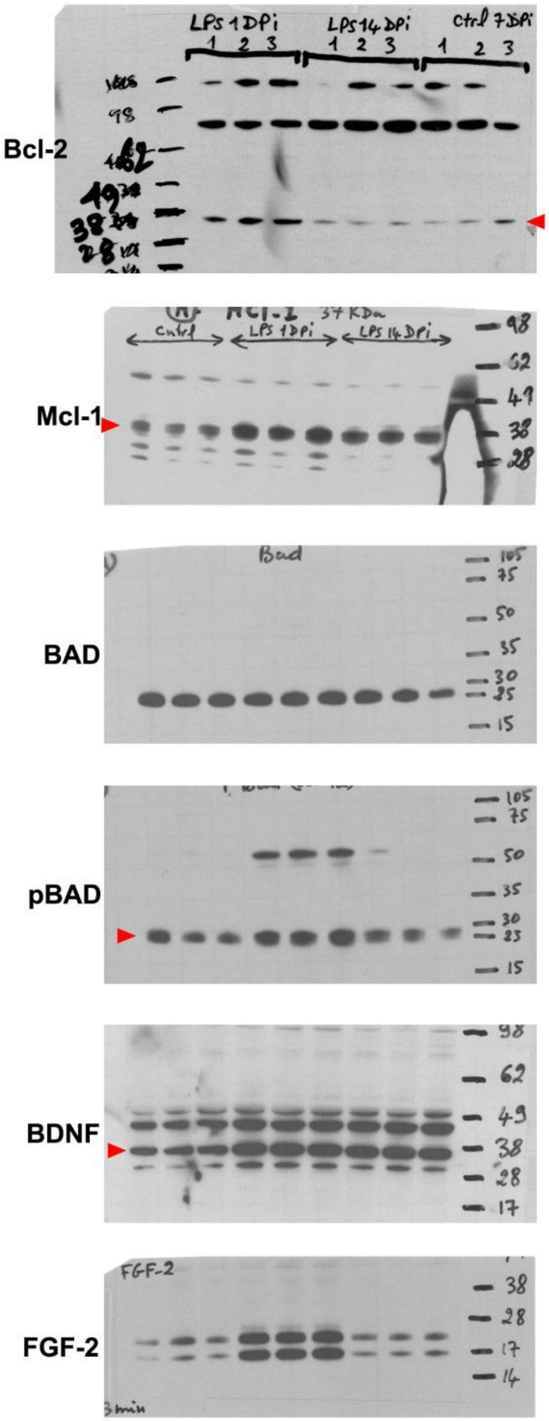


**Supplementary Figure 7 | Full western blots containing bands presented in Figure 5a.**

Arrowheads indicate band of interest if multiple bands appear in a lane.
